# Intercession for Dr. Schoeppe

**Published:** 1869-12

**Authors:** 


### Intercession for Dr. Schoeppe.

Dr. J. Roesing, North German Consul General in this city, states that he has received intelligence that Baron von Gerolt, the North German Minister at Washington, has seen Governor Geary at Harrisburg, Penn., relative to the Dr. Schoeppe case, and that the Governor has declared himself willing to revise the case from a memorandum which is being prepared at the office of the North German Embassy.—*N. Y. Times.*

—:o:—

W. H. Peabody has obtained, for the use of the profession, a supply of the new hypnotic "Chloral," so that those who wish, can make trial of its properties.

